# Post-exercise high-sensitivity troponin T levels in patients with suspected unstable angina

**DOI:** 10.1371/journal.pone.0222230

**Published:** 2019-09-09

**Authors:** Gaetano Antonio Lanza, Erica Mencarelli, Veronica Melita, Antonio Tota, Maurizio Gabrielli, Filippo Sarullo, Chiara Cordischi, Annalisa Potenza, Silvia Cardone, Antonio De Vita, Antonio Bisignani, Laura Manfredonia, Giuseppa Caccamo, Giuseppe Vitale, Silvia Baroni, Mirca Antenucci, Filippo Crea, Francesco Franceschi

**Affiliations:** 1 Fondazione Policlinico Universitario A. Gemelli IRCCS, Università Cattolica del Sacro Cuore, Department of Cardiovascular Disease, Roma, Italy; 2 Fondazione Policlinico Universitario A. Gemelli IRCCS, Università Cattolica del Sacro Cuore, Department of Emergency Medicine, Roma, Italy; 3 Ospedale Buccheri La Ferla, Cardiac Rehabilitation Unit, Palermo, Italy; 4 Fondazione Policlinico Universitario A. Gemelli IRCCS, Università Cattolica del Sacro Cuore, Department of Clinical Chemistry, Roma, Italy; University of Bologna, ITALY

## Abstract

**Background:**

Previous studies showed that troponin blood levels may increase after exercise. In this study we assessed whether, among patients admitted with suspected unstable angina, the increase in high-sensitive troponin T (hs-TnT) levels after exercise stress test (EST) might help identify those with obstructive coronary artery disease (CAD) and predict symptom recurrence during short term follow-up.

**Methods:**

Maximal treadmill EST was performed in 69 consecutive patients admitted to the emergency room with a suspicion of unstable angina (acute chest pain but confirmed normal serum levels of cardiac troponins) was measured before and 4 hours after EST. Coronary angiography was performed in 22 patients (32.8%).

**Results:**

hs-TnT increased after EST compared to baseline in the whole population (from 0.84±0.65 to 1.17±0.87 ng/dL, p<0.001). The increase was similar in patients with positive (n = 14) and negative (n = 55) EST (p = 0.72), and was also similar in patients with (n = 12) and without (n = 10) obstructive CAD at angiography (p = 0.91). The achievement of a heart rate at peak EST ≥85% of that predicted for age was the variable mainly associated with the post-EST hs-TnT increase at multivariable linear regression analysis (p = 0.005). The change after EST of hs-TnT did not predict the recurrence of symptoms or readmission for chest pain at 6-month follow-up.

**Conclusions:**

Our data show that hs-TnT increased after EST in patients with suspected unstable angina, which seemed largely independent of most clinical and laboratory variables. Thus, hs-TnT assessed after EST does not seem to be helpful to identify patients with obstructive CAD in this kind of patients.

## Introduction

The detection of increased blood levels of troponins has become the reference marker for the diagnosis of acute myocardial infarction [1,2]. However, troponins may increase in various conditions of myocardial injury different from ischemic myocardial necrosis [3,4]. Furthermore, some studies showed that troponins may also increase simply following myocardial ischemia, in the absence of cell necrosis [5–11]. The latter data suggested the possibility that a significant increase of troponins after stress testing might increase the diagnostic yield for obstructive coronary artery disease (CAD) [12]. However, other studies found that troponins also increase after physical exertion and other types of stressful stimuli in the absence of obstructive CAD and myocardial ischemia, thus suggesting that mechanisms different from ischemia may also be responsible for their raise following increased myocardial workload [13–18].

All previous studies, however, were conducted in patients with stable forms of definite or suspected CAD, or even in apparently healthy subjects. No previous study, instead, investigated the response to exercise of troponins in patients with a suspicion of acute coronary syndrome (ACS). In these patients the detection of ischemic abnormalities at the electrocardiogram (ECG) and/or increased levels of troponins is usually an indication to admission and invasive management [18–20]. In those with no significant ECG abnormalities and troponin increase, instead, exercise stress testing (EST) is frequently used to guide clinical management [19,20]. The results of EST, however, are suboptimal to identify significant atherothrombotic CAD in this low-risk patients [21].

Thus, the primary aim of this study was to assess whether the changes of troponin serum levels after EST might help identify, among patients admitted with acute chest pain suggesting unstable unstable angina, those with significant atherothrombotic CAD. Furthermore, we assessed whether the post-EST troponin levels might be helpful to identify those with recurrence of symptoms over a 6-month period of follow-up.

## Materials and methods

We studied consecutive patients admitted to the Emergency Department of our hospitals for acute chest pain suspected for NSTE-ACS, showing normal ECG and normal serum levels of routine cardiac troponins on two determinations obtained with an interval of 6 hours (possible unstable angina).

Patients were excluded if they had evidence of any other relevant cardiac or systemic disease, increased creatinine serum levels or ECG abnormalities that precluded a careful assessment of ST-segment changes. Patients were also excluded if they had a history of previous acute myocardial infarction, coronary by-pass surgery or percutaneous coronary intervention (PCI) with incomplete revascularization. Patients were included, however, in case of previous PCI with documentation of complete revascularization.

The presence of cardiovascular risk factors was carefully assessed and drug therapy recorded. Hypertension was defined as the detection of blood pressure (BP) ≥140/90 mmHg or use of anti-hypertensive drugs. Hypercholesterolemia was defined as total cholesterol levels >220 mg/dL or use of anti-cholesterolemic drugs. Diabetes mellitus was defined as the detection of use of antidiabetic drugs or a glycated hemoglobin >6.5%. Active smoking was defined as having smoked any cigarettes in the last 6 months.

The study protocol was approved by the Review Boards of our Institutions (Università Cattolica del Sacro Cuore, Rome, and Ospedale Buccheri-La Ferla, Palermo; Italy) and a written informed consent to participate in the study was always obtained from patients.

All patients underwent a symptom/sign-limited Bruce treadmill EST under continuous ECG monitoring as soon as possible after the second determination of serum troponin was available. EST was considered positive for myocardial ischemia when a horizontal or downsloping ST-segment depression ≥1 mm or an upsloping ST-segment depression ≥1.5 mm at 0.08 s from the J point was detected.

Criteria for EST interruption included physical exhaustion, worsening symptoms (angina, dyspnea), occurrence of any potentially dangerous clinical condition (e.g., pre-syncope, hypotensive or hypertensive response, arrhythmias), ST-segment elevation ≥1 mm or ST-segment depression ≥3 mm in two or more contiguous leads.

The clinical management of the patients following EST was left at the total discretion of the attending physicians and consultant cardiologist of the emergency department. Blood samples were collected immediately before and 4 hours after peak EST. Samples were centrifuged at 2800 rpm for 20 min, and serum aliquots of 1.5 mL were stored for subsequent analyses. hs-TnT levels were measured after the completion of the study using a high sensitivity electro-chemio-immune-luminescence assay (ECLIA method, Roche Italia, Monza, Italy), with the 99^th^ URL percentiles being 1.4 ng/dL. Accordingly, hs-TnT levels measured immediately before and 4 hours after exercise were unknown to the clinicians and, therefore, in no case could be used to guide patients’ management.

When indicated by caring physicians, coronary angiography was performed by radial or femoral artery access using standard procedures. Significant CAD was defined as the presence of a ≥50% diameter stenosis or evidence, with the help of intracoronary ultrasound or optical coherence tomography, of thrombotic lesions in any epicardial coronary vessel.

A clinical follow-up was conducted at 6 months by on-site visit or telephone interview. Clinical end-points included death, recurrence of chest pain and re-admission for acute chest pain.

Statistical analyses were performed by SPSS 21.0 statistical software (SPSS Inc., Florence, Italy). The Kolmogorov-Smirnov test was applied to assess whether continuous variables had a distribution significantly different from the normal one. Since both basal and post-EST hs-TnT levels showed a non-normal distribution, a logarithmic transformation of values was done and all statistical analyses were done by parametric tests using logarithmic values of hs-TnT concentrations, although raw data are shown in the text. Between-group comparisons of baseline continuous variables were done by independent t-test, whereas proportions were compared by Fisher exact test. The influence of variables on the EST-induced changes of hs-TnT was first assessed by two-way repeated measure analysis of variance (ANOVA). To this aim, continuous variables were dichotomized according to pre-specified cut-off values, including: 1) age ≥65 vs. <65 years; 2) HR at peak EST ≥85% vs. <85% of maximal HR predicted for age; 3) systolic BP at peak EST >median vs. ≤median value. In case of global statistical significance post-hoc intra-group comparisons were done by paired t-test. Multivariable linear regression was applied to identify variables independently associated with the increase of hs-TnT after EST, including in the models only variables showing a significant or borderline statistical association (p≤0.1) with the increase of hs-TnT at univariable analysis. Data are reported as means with standard deviations or proportions unless differently indicated. A p<0.05 was always required for statistical significance.

## Results

Overall, 69 patients were enrolled in the study. The main clinical characteristics of the patients are summarized in Table 1. Interestingly, 6 patients (8.7%), who had confirmed normal levels of ultra-sensitive TnI at the routine clinical assessment, showed increased hs-TnT serum levels at baseline (>1.4 ng/dL).

**Table 1:** Main clinical data of the 69 patients included in the study.

Age (years)	58±10
Sex (male)	47 (68%)
*Cardiovascular risk factors*	
Family history of CAD	9 (13%)
Hypertension	46 (567%)
Hypercholesterolemia	30 (44%)
Diabetes mellitus	10 (15%)
Active smoking	15 (22%)
Previous PCI	14 (20%)
*Drug therapy on admission*	
Beta-blockers	17 (25%)
Ca-channel blockers	7 (10%)
Nitrates	2 (3%)
ACE inhibitors/ARBs	30 (44%)
Diuretics	10 (14%)
Aspirin	24 (35%)
Statins	22 (32%)
Oral antidiabetic drugs	10 (14%)

ACE = Angiotensin converting enzyme; AMI = acute myocardial infarction; ARBs = Angiotensin II receptor blockers; PCI = percutaneous coronary intervention.

**Fig 1:**
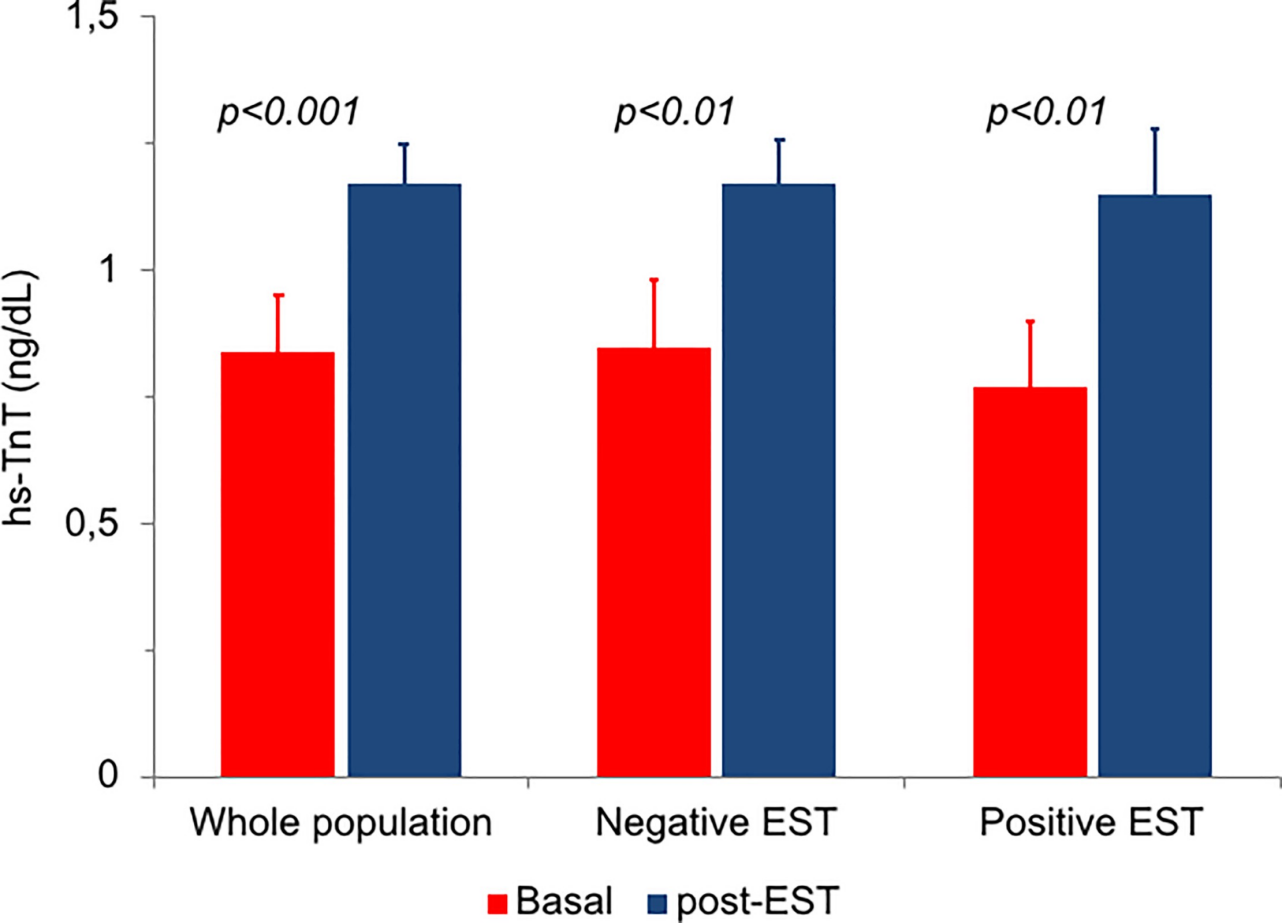
Changes in hs-TnT concentration at baseline and after 4 hours from symptom/sign-limited EST in the whole population of patients (n = 69) and in the groups of patients with positive (n = 14) and negative (n = 55) EST. The difference in the change between the latter 2 groups was not statistically significant (p = 0.72). Data are means with standard errors.

Serum hs-TnT concentrations increased 4 hours after EST compared to baseline in the whole population (from 0.84±0.65 to 1.17±0.87 ng/dL, p<0.001) (Fig 1). Overall, 14 patients (20.3%) had hs-TnT >1.4 ng/dL.

The main clinical characteristics of patients and the results of EST are summarized in Table 2. EST was positive in 14 patients (20.3%) and negative in 55 (79.7%). There were no significant differences between the 2 groups in EST duration as well as HR and BP at peak EST. Basal hs-TnT levels were comparable in the 2 groups. Moreover, a similar increase in hs-TnT after EST (p<0.01 for both) was observed in patients with positive (from 0.82±0.48 to 1.20±0.47 ng/dL) and negative (from 0.85±0.69 to 1.17±0.95 ng/dL) EST (p for changes = 0.72) (Fig 1).

**Table 2:** Main results of exercise stress test.

	Positive EST (n = 14)	Negative EST (n = 55)	p
*Basal*			
Systolic BP, mmHg	119±13	116±13	0.43
Diastolic BP, mmHg	77±8	72±10	0.10
HR (bpm)	71±10	74±13	0.56
*Peak exercise*			
Exercise duration, s	457±147	473±165	0.74
Systolic BP, mmHg	158±14	159±24	0.94
Diastolic BP, mmHg	84±10	84±8	0.98
HR (bpm)	134±14	138±21	0.54
Predicted HR, %	84±10	84±12	0.95
ST max (mm)	1.43±0.76	-	-
*hs-TnT assessment*			
Pre-EST hs-TnT	0.82±0.48	0.85±0.69	0.67
Post-EST hs-TnT	1.20±0.47	1.17±0.95	0.95
hs-TnT increase, ng/dL	0.39 ±0.33	0.31 ±0.72	0.72
hs-TnT increase, %	80.4±92	45.4 ±76	0.15

BP = blood pressure; EST = exercise stress test; hs-TnT = high-sensitive troponin T;; HR = heart rate.

**Fig 2:**
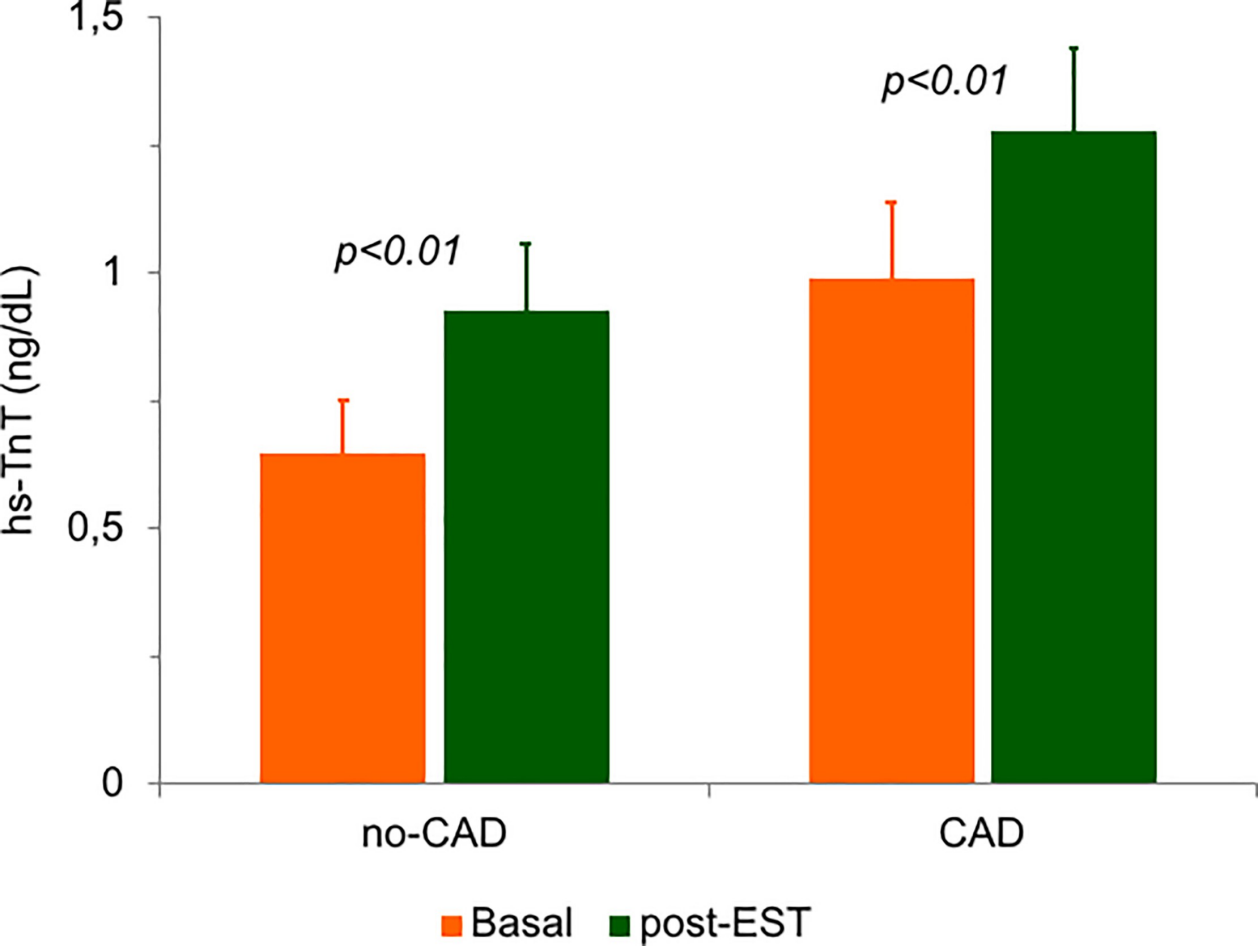
Changes in hs-TnT concentration at baseline and after 4 hours from symptom/sign-limited EST in the groups of patients with (n = 12) and without (n = 10) obstructive stenosis of coronary arteries at angiography. The difference in the change between the latter 2 groups was not statistically significant (p = 0.91). Data are means with standard errors.

Overall, 22 patients (32.8%) were referred for coronary angiography by the attending clinicians, including all 14 patients with positive EST and 8 out of 55 patients (14.6%) with negative EST. The reason for indication to coronary angiography despite negative EST in the latter 8 patients was a persisting suspect by the attending physicians of an ischemic origin of the index chest pain, based on clinical characteristics, particularly in patients with a previous history of PCI. Indeed, 6 out of these 8 patients (75%) had undergone a previous PCI vs. only 5 out of 47 patients (10.6%) with negative EST who were discharged without undergoing coronary angiography (p<0.01). Obstructive CAD was found in 12 patients (54.5%), 7 with positive EST (50%) and 5 with negative EST (62.5%; p = 0.68). Basal hs-TnT tended to be higher in patients with compared to those without obstructive CAD (0.99±0.47 vs. 0.65±0.35 ng/dL; p = 0.055). However, a similar increase in hs-TnT (p<0.01 for both) was found in the 2 groups after EST (to 1.28±0.51 and to 0.93±0.44 ng/dL, respectively; p for changes = 0.91; Fig 2).

The relation of the changes in hs-TnT serum levels after EST with the main clinical and EST variables is shown in Table 3. The increase in hs-TnT was independent of age, sex and cardiovascular risk factors. However, no significant changes were observed in the small group (n = 9) of diabetic patients, whereas a higher significant increase in hs-TnT was observed in patients with hypertension (vs. no hypertension).

**Table 3:** Changes in hs-TnT with EST in various subgroups of patients.

		Basal hs-TnT (ng/dL)	Post-EST hs-TnT (ng/dL)	P
*Clinical variables*				
Age ≥65 years	Yes (n = 17) No (n = 52)	1.12 ±0.85 0.75±0.55	1.69±1.09 1.01 ±0.72	0.09
Sex	Male (n = 47) Female (n = 22)	0.88±0.60 0.73±0.70	1.30±0.97 0.88±0.48	0.13
Hypertension	Yes (n = 46) No (n = 23)	0.89±0.61 0.76±0.72	1.35±0.97* 0.83±0.49*	0.016
Hypercholesterolemia	Yes (n = 29) No (n = 40)	0.99±0.88 0.74±0.39	1.38±0.98* 1.02±0.76*	0.21
Smoking	Yes (n = 15) No (n = 54)	0.65±0.31 0.90±0.71	1.04±1.08 1.21±0.81	0.72
Diabetes	Yes (n = 10) No (n = 59)	1.24 ±0.90 0.78±0.58	1.20±0.44* 1.17±0.93*	0.05
Previous PCI	Yes (n = 14) No (n = 55)	1.11 ±0.99 0.78±0.52	1.27 ±1.10 1.15±0.82	0.28
*EST variables*				
EST end-stage	I or II (n = 13)* III (n = 29) IV (n = 26)	1.28±1.20 0.81±0.38 0.65±0.29	1.40±1.07 1.19±0.81 1.03±0.83	0.60
HRpeak ≥85%	Yes (n = 36) No (n = 33)	0.72±0.33 0.98±0.86	1.24±0.92* 1.10 ±0.83*	0.012
Systolic BPpeak >150	Yes (n = 32) No (n = 37)	0.75±0.33 0.92±0.83	1.25±0.97* 1.11 ±0.79*	0.08
*Follow-up*				
Recurrent CP	Yes (n = 23) No (n = 46)	0.96±0.73 0.79±0.60	1.18±0.83 1.17±0.90	0.33
Readmission for CP	Yes (n = 6) No (n = 63)	0.80±0.54 0.85±0.66	1.07±0.67 1.18±0.89	0.81

*Only 1 patient stopped at the end of the I stage. †Data on 22 patients who underwent coronary angiography. CAD = coronary artery disease; CP = chest pain; EST = exercise stress test; EST = exercise stress test; PCI percutaneous coronary intervention.

The increase in hs-TnT also occurred independently of the level of exercise and the maximal HR and systolic BP achieved during the test. However, hs-TnT showed a greater increase after EST in patients who achieved an HR ≥85% (vs. <85%) of the maximal value predicted for age.

The results of multivariable analyses are summarized in Table 4. Maximal HR ≥85% of the value predicted for age was the only variable significantly associated with the hs-TnT increase after EST.

**Table 4:** Variables independently associated with greater increase of hs-TnT after exercise stress test at multiple linear regression analysis.

	β coefficient	95% confidence lomits	p
Age >65 years	-0.026	-0.252, 0.200	0.82
Hypertension	0.171	-0.033, 0.374	0.099
Diabetes mellitus	-0.251	-0.510, -0.007	0.056
EST peak HR ≥85%	0.283	0.087, 0.479	0.005
Systolic BP >150 mmHg	0.058	-0.132, 0.247	0.55

BP = blood pressure; EST = exercise stress test; HR = heart rate.

During 6-month follow-up no major clinical event occurred. Overall, 23 patients (33.3%) reported recurrence of chest pain and 6 patients (8.7%) were readmitted to hospital for acute chest pain. Both basal hs-TnT and hs-TnT changes after exercise did not differ between patients with or without symptoms at follow-up (Table 3).

## Discussion

This is the first study that assessed whether the changes in cardiac troponins after an EST in patients with a suspicion of unstable angina may be helpful to identify those who actually have atherothrombotic disease and/or are at increased risk of events during short-term follow-up.

Our data show that hs-TnT increased significantly after EST in patients with a clinical presentation of suspected unstable angina, but the increase was independent of the evidence of myocardial ischemia at EST and obstructive CAD at angiography, thus suggesting that it was largely related to exercise in itself. Of note, the changes did not also predict the recurrence of chest pain over a follow-up period of 6 months.

The increase of troponins after exercise was reported in several previous studies that involved clinically stable patients and apparently healthy subjects but showed some discordant results. Some studies, indeed, reported a greater increase of troponins in ischemic, compared to non-ischemic, patients [5,8,11], but other studies failed to demonstrate a significant relation with both myocardial ischemia and presence of obstructive CAD [17,18]. Our study shows results that are in agreement with those of the latter reports, as no significant relation with both ischemic ECG changes and obstructive CAD could be found.

A practical consequence of our data is that, in patients with chest pain suspected for an acute coronary syndrome who show a small increase of serum hs-TnT levels, it is mandatory to exclude, together with other non-ischemic conditions able to increase troponin levels [3,4,22,23], significant efforts in the previous hours. The troponin raise might indeed be a mere consequence of a previous physical activity, thus leading to a misinterpretation of the increase as a marker of acute ischemic damage. In our study, indeed, 8 out of 63 patients (12.7%) with basal hs-TnT serum levels in the normal range showed values after EST above the threshold for a diagnosis of “acute myocardial infarction”.

It is worth noting that the increase of hs-TnT in our patients was also largely independent of various clinical and EST variables, although, at univariable analysis, it was found greater in hypertensive patients and in patients who during EST achieved a maximal HR ≥85% of that predicted for age, while no significant changes were detected in diabetic patients. The greater hs-TnT increase after exercise in patients achieving higher HR and in hypertensive patients can be attributed to the high cardiac workload and a likely increased left ventricle mass [17], respectively. On the other hand, the lack of changes in hs-TnT after EST in diabetic patients does not have ready explanations; however, the diabetes subgroup included 10 patients only, and therefore further assessment is required to verify whether the different results observed in this subgroup, as compared to all others, was real or just an effect of chance.

Importantly, however, at multivariable linear regression analyses, however, only HR at peak EST emerged as the variable that independently influenced the EST-related increase of hs-TnT, thus suggesting that a higher cardiac myocardial oxygen consumption and work played a major role in the increased levels of hs-TnT with exercise.

### Limitations of the study

Some limitations of our study should be acknowledged. First, the number of patients included in the study (in particular, those with available coronary angiography results) was rather small, and therefore our data should be considered as explorative and confirmed in larger populations. Second, we measured hs-TnT after 4 hours from exercise only; thus, we cannot exclude that a higher proportion of patients might have displayed increased hs-TnT levels if other blood samples had been obtained later (e.g., at 6–12 hours); unfortunately, this was not possible as patients not admitted to hospital for further assessment had to be discharged as soon as possible. Moreover, for the same reason, we could not obtain multiple samples from patients after EST; thus, whether the curve of the changes in hs-TnT concentrations over time, derived from multiple blood samples, might be helpful to identify patients with obstructive CAD remains to be established. Third, the diabetic status of patients was only assessed on admission by antidiabetic therapy and measurement of glycated hemoglobin; a careful assessment of the glycemic status could not be performed in all patients, indeed, due to the early discharge of most of them; on the other hand, the classification of smoking status we applied was rather arbitrary; accordingly, further studies are required to better clarify the effects of diabetes and smoking status on the troponin changes after EST in patients admitted with acute chest pain. Finally, at the time of admission some patients were taking anti-ischemic drugs that could not be withdrawn; thus, also whether some form of treatment may influence the response to EST of troponin(s) needs to be clarified in appropriate studies.
